# Osteochondral Cell Fate in the Mandibular Condyle: A Shared Fate Decision Across Development, Degeneration, and Regeneration

**DOI:** 10.3390/cells15201913

**Published:** 2026-10-21

**Authors:** Lingfeng Xu, Meiqing Wang

**Affiliations:** Shanghai Key Laboratory of Craniomaxillofacial Development and Diseases, Fudan University, Shanghai 200001, China; xulingfeng_dentist@aliyun.com

**Keywords:** cell fate, developmental signalling, mandibular condyle, mechanical loading, osteoarthritis, temporomandibular joint (TMJ)

## Abstract

**Highlights:**

**What are the main findings?**

- Mechanical loading links condylar development, degeneration and regeneration.
- PTHrP, IHH and Wnt/β-catenin signalling coordinate osteochondral cell fate.

**What is the implication of the main finding?**

- Repair may require restoring loading and cell-fate balance.

**Abstract:**

The mandibular condyle is highly responsive to mechanical loading, which shapes its development and maintains osteochondral homeostasis. Understanding how loading regulates osteochondral cell fate may connect developmental mechanisms with strategies to limit mechanically driven degeneration and promote repair. In this narrative review, we integrate evidence across development, degeneration, and regeneration into a framework centred on a shared cell-fate decision: coordination between progenitor renewal, chondrocyte maturation, and endochondral bone formation. We examine the reported dual modes of parathyroid hormone-related protein (PTHrP) signalling and their interactions with Indian hedgehog (IHH) and canonical Wnt/β-catenin signalling within the broader network governing this balance. Physiological loading supports the spatial and temporal coordination of these pathways, whereas persistent abnormal loading may accelerate hypertrophic differentiation and impair progenitor replenishment, contributing to cartilage damage and aberrant osteochondral remodelling. Effective regeneration may therefore depend on restoring both a favourable mechanical environment and coordinated cell-fate regulation. Accordingly, where pathological occlusal loading contributes to degeneration, correcting the mechanical environment may merit consideration alongside biological interventions tailored to the joint’s residual repair capacity. Potential approaches include pathway modulation to support responsive endogenous cells and cell supplementation when progenitor reserves are insufficient.

## 1. Introduction

Osteochondroprogenitors are heterogeneous skeletal precursors that generate both chondrogenic and osteogenic lineages [1,2,3]. By producing chondrocytes and osteoblast-lineage cells, these populations contribute to the formation, growth, and maintenance of cartilage and bone [3,4,5]. Cartilage and bone form an integrated skeletal system that provides structural support and transmits mechanical loads [6,7]. The ability to bear and transmit loads depends not only on the architecture and integration of cartilage and bone, which are established during development through molecular signals that regulate cell proliferation, differentiation and matrix formation [8,9,10], but also on the forces acting on these tissues [11,12]. Both developmental and mechanical inputs act on overlapping signalling networks that coordinate cell fate, tissue maturation and tissue remodelling [2,13,14]. The shape and arrangement of skeletal tissues emerge during development, while mechanical loading influences how these tissues grow. Furthermore, functional loading helps shape developing tissues and subsequently maintains their structure and function. Thus, development and mechanical adaptation are closely intertwined.

In the temporomandibular joint (TMJ), the mandibular condyle and articular fossa continue to develop throughout childhood and adolescence as the permanent teeth erupt and occlusion is established [15,16,17]. The initially shallow articular fossa gradually becomes more concave [15,16,18]. Growth and remodelling of the mandibular condylar cartilage, together with the associated increase in condylar volume, are regulated by genetic factors and modulated by functional loading. Mechanical forces generated during mastication influence cell proliferation, differentiation, and matrix production, thereby shaping cartilage thickness and subchondral bone formation [19,20,21,22,23,24]. Accordingly, abnormal loading associated with malocclusion may interfere with the normal development of the joint [25]. Although developmental growth declines after adolescence, the mandibular condyle retains some capacity for load-responsive remodelling and can therefore adapt to changes in the dentition and occlusal environment, including tooth loss, restorative treatment, orthodontic treatment and orthognathic surgery [15,26].

Standard laboratory cages provide limited space for movement and reduce spontaneous locomotor activity in mice [27,28]. The cages are therefore likely to narrow the range of activity-related mechanical loading experienced by the limbs. Although limb models remain valuable for studying the genetic regulation of skeletal development, responses observed under standard housing conditions may not fully reflect those produced by more diverse natural movements [29,30]. Masticatory loading, by contrast, occurs routinely under standard housing conditions and can be experimentally manipulated by altering diet consistency or occlusion [31,32]. Experimental manipulation of dental occlusion therefore provides a useful approach for studying TMJ articular mechanobiology.

**Figure 1:**
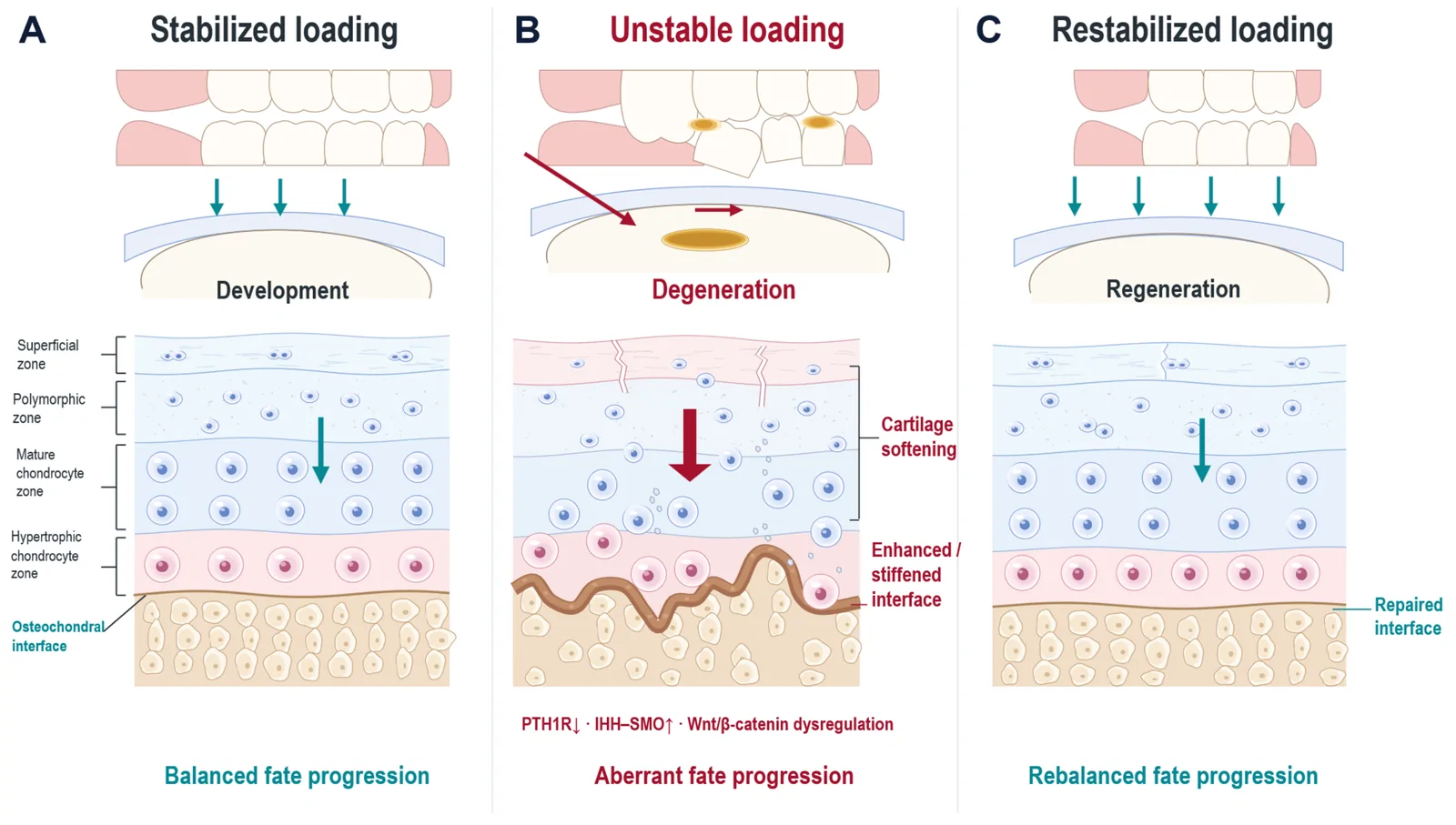
Load-sensitive coordination of osteochondral cell fate during condylar development, degeneration and repair. (**A**) Functional loading supports the coordination of superficial-zone progenitor maintenance, chondrocyte maturation and subchondral bone formation. (**B**) Abnormal loading is associated with accelerated terminal differentiation, loss of superficial-zone cells, cartilage softening and stiffening of the osteochondral interface. The signalling changes shown beneath panel B summarise reduced PTH1R signalling, enhanced IHH–SMO activity and dysregulated Wnt/β-catenin signalling in the experimental models discussed in the text. (**C**) Restoration of a compatible loading environment is proposed to support tissue repair and renewed coordination of osteochondral cell-state progression. Arrows above the cartilage indicate loading, whereas arrows within the cartilage indicate progression towards deeper cell states. The columns provide a conceptual synthesis of developmental and experimental disease and repair findings. PTH1R, parathyroid hormone 1 receptor; IHH, Indian hedgehog; SMO, Smoothened.

The coordinated regulation of osteochondral cell fate is crucial for condylar development and tissue maintenance. Loss of this coordination can promote degeneration, whereas its restoration may facilitate regeneration. Development, degeneration, and regeneration of the mandibular condyle have been studied extensively, but mostly as separate processes [33,34,35]. Evidence from these fields has rarely been integrated to explain how changes in osteochondral cell-fate regulation link the three processes. In this narrative review, we integrate evidence on developmental processes and functional loading to examine how progenitor renewal, chondrocyte maturation, and bone formation are coordinated in the mandibular condyle. We propose that development establishes this coordination, degeneration disrupts it, and regeneration depends on its restoration, with modulation of mechanical loading playing a critical role in all three processes (Figure 1).

## 2. Postnatal Osteochondral Development and Loading-Dependent Growth of the Mandibular Condyle

The mandibular condylar cartilage is a cranial neural crest-derived secondary cartilage [36,37]. During postnatal growth, progenitor-like cells are retained beneath the articular surface, particularly within the superficial zone and polymorphic zone. Lineage-tracing studies using *αSMA*-CreERT2 (α-smooth muscle actin) [38], *Scx*-CreERT2 (scleraxis) [39] and *Gli1*-driven inducible Cre lines [40,41], combined with permanent lineage reporters, indicate that progenitor-like cells in the superficial zone and polymorphic zone generate chondrocytes during postnatal condylar growth. The descendants of the superficial-zone progenitors populate the deeper zones and stay at different stages of differentiation [38,42,43], forming, from the articular surface towards the subchondral bone, the superficial zone, polymorphic zone, mature chondrocyte zone and hypertrophic chondrocyte zone [42,43,44,45]. As cells progress through maturation and hypertrophic differentiation across these zones, matrix mineralisation and endochondral ossification contribute to subchondral bone formation [4,42,46]. Matrix composition varies by zone. Type I collagen is most abundant in the superficial zone, whereas type II collagen and aggrecan are predominant in the mature chondrocyte zone. Type X collagen is expressed by hypertrophic chondrocytes in the hypertrophic chondrocyte zone [47,48]. Lineage tracing with tamoxifen-inducible *Acan*-CreERT2 and constitutive *Col10a1*-Cre drivers shows that chondrocyte-derived cells can give rise to osteoblasts and osteocytes in the condylar neck and subchondral bone [4]. In this way, chondrocytes participate in endochondral bone formation during postnatal growth [36,42,49]. With skeletal maturation, progenitor proliferation and endochondral growth decline but do not cease, while tissue maintenance and remodelling become more prominent [42,50].

Sufficient functional loading is necessary for the growth of the mandibular condyle. Functional loading is modified by dental occlusion. It has been reported that growing rabbits fed a soft diet had thinner condylar cartilage than those fed a solid diet [51]. Soft-diet mice also had narrower condyles and lower bone volume than mice fed a hard diet [52]. Soft-diet feeding reduced chondrocyte proliferation and maturation, as well as mandibular growth [53]. Lineage-tracing studies using *Acan*-CreERT2 and *Col10a1*-Cre showed fewer chondrocyte-derived bone cells in soft-diet mice than in hard-diet controls [53]. In growing mice, combined incisor trimming and soft-diet feeding reduced chondrocyte maturation, decreased condylar cartilage thickness, and transiently impaired subchondral bone development [54].

Aberrant occlusion can alter the functional loading environment of the developing TMJ. In a mouse model of occlusion-induced mandibular deviation, masseter muscle activity is decreased, and condyle size and mandibular bone mineral density are reduced [55]. In growing rats subjected to experimental unilateral anterior crossbite (UAC), a model of pathological occlusal loading that induces TMJ degeneration, hypertrophic differentiation was disrupted, leading to increased abnormal mineral deposition in the deep condylar cartilage [25,56]. The detrimental effects of abnormal occlusion may also be modulated by masticatory function. In growing mice with UAC, animals fed large food pieces showed greater cartilage and subchondral bone loss than those fed small pieces [57]. Normal postnatal condylar development therefore depends on adequate masticatory stimulation and balanced loading that can be transmitted normally to the condyle. Although direct clinical evidence in children remains limited, animal studies suggest that both insufficient stimulation, as occurs with a soft diet, and maladaptive loading associated with aberrant occlusion can disturb normal development of the mandibular condyle.

## 3. Developmental Signalling in Load-Responsive Osteochondral Cell-State Transitions in the Mandibular Condyle

### 3.1. Dual Modes of PTHrP Signalling

In the mandibular condyle, parathyroid hormone-related protein (PTHrP) coordinates the supply of new chondrocytes with their progression towards hypertrophic differentiation. Cells expressing *Pthlh*, the gene encoding PTHrP, are mainly concentrated in the superficial zone, and the secreted PTHrP protein diffuses through the underlying polymorphic zone and mature chondrocyte zone [58]. Analysis of PTHrP-deficient mice shows that loss of *Pthlh* reduces the proliferation of runt-related transcription factor 2 (RUNX2)-positive chondrogenic precursors in the polymorphic zone. It also induces premature hypertrophic differentiation, followed by depletion of the chondrocyte population. These findings indicate that PTHrP maintains a continuous supply of new chondrocytes by supporting precursor proliferation and restraining premature hypertrophy [58].

PTHrP exerts its biological effects through two partly distinct modes. In the receptor-dependent mode, its N-terminal region binds the parathyroid hormone 1 receptor (PTH1R), which is expressed mainly in the mature and hypertrophic chondrocyte zones of the developing condyle [59,60]. PTHrP binding to PTH1R activates the stimulatory G-protein α-subunit (Gαs)–cyclic adenosine monophosphate (cAMP)–protein kinase A (PKA) pathway, thereby delaying hypertrophic and terminal differentiation. In growth-plate chondrocytes, this effect involves the nuclear retention of histone deacetylases 4 and 5 (HDAC4/5), which suppress myocyte enhancer factor 2C (MEF2C)–RUNX2-dependent hypertrophic transcription [61,62]. PTHrP can also act through its nuclear localisation segment (NLS). This region mediates the nuclear entry of PTHrP, thereby promoting cell proliferation through an intracrine mechanism [63,64]. Although the effects of these two modes may overlap, current evidence suggests that they make distinct contributions to chondrocyte production and maturation [58]. PTH1R signalling mainly restrains premature hypertrophic differentiation, whereas signalling mediated by the PTHrP nuclear localisation segment (PTHrP-NLS) is mainly associated with the proliferation of cells in the superficial zone.

### 3.2. IHH Signalling and Its Relationship with PTHrP Signalling

During embryonic development, *Ihh* is strongly expressed in mature and prehypertrophic chondrocytes at embryonic days 15.5 and 16.5 (E15.5 and E16.5) and remains localised near the mature-to-hypertrophic transition at E18.5 [49,58,65]. This spatial pattern persists postnatally, with *Ihh* expression concentrated in prehypertrophic and early hypertrophic chondrocytes [33,66]. In the absence of Indian hedgehog (IHH), patched 1 (PTCH1) inhibits Smoothened (SMO). IHH binding relieves this inhibition, enabling SMO in the primary cilium to regulate GLI2/3 activity and initiate GLI-dependent transcription [67,68,69,70]. GLI2 functions as the principal initial transcriptional activator, whereas Hedgehog signalling induces *Gli1* transcription, with the resulting GLI1 protein acting mainly as a downstream transcriptional amplifier [70,71]. Because *Ptch1* and *Gli1* are Hedgehog-responsive genes, their expression and reporter activity serve as readouts of pathway activation. Notably, *Ptch1* expression and *Gli1* reporter activity are detected mainly in the polymorphic and mature chondrocyte zones. This indicates that cells in these zones respond to IHH produced in the mature and hypertrophic chondrocyte zones postnatally [49,66]. In brief, IHH produced by deeper chondrocytes acts on cells in the superficial zone, where it supports progenitor cell proliferation and maintains the orderly spatial progression of chondrocyte maturation [42,49,66].

Postnatal conditional deletion studies confirm the continued requirement for IHH. In *Ihh*-deficient mouse embryos, cell proliferation in the polymorphic zone and PTHrP expression are markedly reduced. The condylar cartilage is consequently smaller and poorly organised, with an expanded hypertrophic chondrocyte zone [49]. In neonatal *Col2a1*-CreER-mediated and juvenile *Acan*-CreERT2-mediated *Ihh* deletion models, loss of IHH disrupted the zonal organisation of the condylar cartilage, reduced the number and proliferation of cells in the superficial and polymorphic zones, decreased the expression of SRY-box transcription factor 9 (SOX9), type II collagen and aggrecan, and led to ectopic chondrocyte hypertrophy near the articular surface [42,66]. One possible explanation is that *Ihh* deficiency reduces PTHrP expression in the embryonic condyle, suggesting that IHH contributes to the maintenance of PTHrP production [49]. Loss of PTHrP also alters *Ihh* expression, but this effect varies across developmental stages. In PTHrP-deficient mice, at E15.5, the *Ihh*-expressing region expands within the mandibular condylar cartilage. However, at E18.5, the *Ihh*-expressing area is markedly reduced [58]. The early expansion is consistent with premature chondrocyte differentiation, whereas the later reduction probably reflects a decline in the supply of cells that progress to the IHH-producing stage. The decline in mature chondrocytes could result from reduced signalling through both PTHrP modes. Beyond this PTHrP-dependent restraint on hypertrophic differentiation, evidence from growth-plate cartilage indicates that IHH can promote hypertrophic differentiation independently of PTHrP, potentially through bone morphogenetic protein (BMP) and canonical Wnt/β-catenin signalling pathways [72]. Together, PTHrP and IHH link progenitor proliferation near the articular surface to chondrocyte differentiation and hypertrophy in the mature and hypertrophic chondrocyte zones. IHH helps maintain PTHrP expression, whereas PTHrP maintains the pool of chondrocytes that subsequently produce IHH.

Detailed reviews discuss PTHrP signalling in condylar development and disease [73] and IHH signalling in TMJ formation [33].

### 3.3. Stage-Dependent Canonical Wnt/β-Catenin Signalling

Canonical Wnt/β-catenin signalling regulates osteochondral cell identity in the mandibular condyle in an activity-level-dependent manner. In the absence of Wnt ligands, the destruction complex, which includes the scaffold protein AXIN, adenomatous polyposis coli protein (APC), and glycogen synthase kinase-3β (GSK3β), promotes β-catenin degradation, thereby limiting T-cell factor/lymphoid enhancer-binding factor (TCF/LEF)-mediated transcription. Wnt binding to Frizzled receptors and low-density lipoprotein receptor-related proteins 5 and 6 (LRP5/6) co-receptors stabilises β-catenin. Stabilised β-catenin enters the nucleus, where it interacts with TCF/LEF transcription factors to regulate target gene expression [74]. In the superficial zone of mandibular condylar cartilage, dickkopf-related protein 3 (DKK3) secreted by leucine-rich repeat-containing G protein-coupled receptor 5 (LGR5)-positive secretory cells and sclerostin produced by chondrocytes create a Wnt-inhibitory environment that restrains pathway activity [75]. Excessive Wnt activation disrupts this state. Constitutive β-catenin activation in S100a4/FSP1-Cre-expressing superficial-zone progenitors (FSP1, fibroblast-specific protein 1) reduces both S100 calcium-binding protein A4 (S100A4)-positive progenitor and SOX9-positive chondroprogenitor populations, ultimately resulting in surface disruption and cartilage loss [76]. During postnatal development, *Axin2* expression, a readout of canonical Wnt/β-catenin activity, progressively decreases in the superficial zone, whereas the number of S100A4-positive progenitors increases. Nevertheless, *Axin2* expression remains detectable in a subset of S100A4-positive cells, indicating that canonical Wnt activity is reduced rather than completely absent in the superficial zone [76]. Together, these findings define the superficial zone as a Wnt-restrained environment in which excessive pathway activation compromises the maintenance of superficial-zone progenitor and early chondrogenic identities.

β-catenin displays a zone-specific distribution in mandibular condylar cartilage, with low levels in the superficial zone and higher levels in the mature and hypertrophic chondrocyte zones [40]. Lineage tracing identifies *Gli1*-lineage cells in the superficial zone as progenitors that contribute to deeper cartilage layers. *Gli1*-CreERT2-mediated deletion of *Ctnnb1*, the gene encoding β-catenin, shows that β-catenin is required for these cells to progress towards hypertrophic differentiation. *Ctnnb1* deletion in this lineage reduces both the number of type X collagen-positive hypertrophic chondrocytes and the thickness of the hypertrophic chondrocyte zone [40]. *Acan*-CreERT2-mediated deletion of *Ctnnb1* similarly reduces cell proliferation and chondrocyte differentiation, resulting in a smaller condylar head, a shorter mandibular ramus and impaired subchondral bone formation. This deletion also reduces the number of *Acan*-lineage bone cells in the subchondral region [77]. *Col10a1*-Cre-mediated deletion of *Ctnnb1* in hypertrophic chondrocytes causes hypertrophic cartilage to accumulate but markedly reduces subchondral trabecular bone and the expression of RUNX2, sclerostin, and dentin matrix acidic phosphoprotein 1 (DMP1) [77]. Conversely, constitutive β-catenin activation accelerates chondrocyte maturation and osteogenic differentiation but also results in disorganised cartilage and structurally immature bone [77,78]. These findings indicate that β-catenin has stage-dependent roles in condylar development. Its activity is limited in the superficial zone but is required for hypertrophic maturation and the subsequent osteogenic differentiation of chondrocyte-derived cells. Both insufficient and excessive β-catenin activity disrupt this progression.

**Figure 2:**
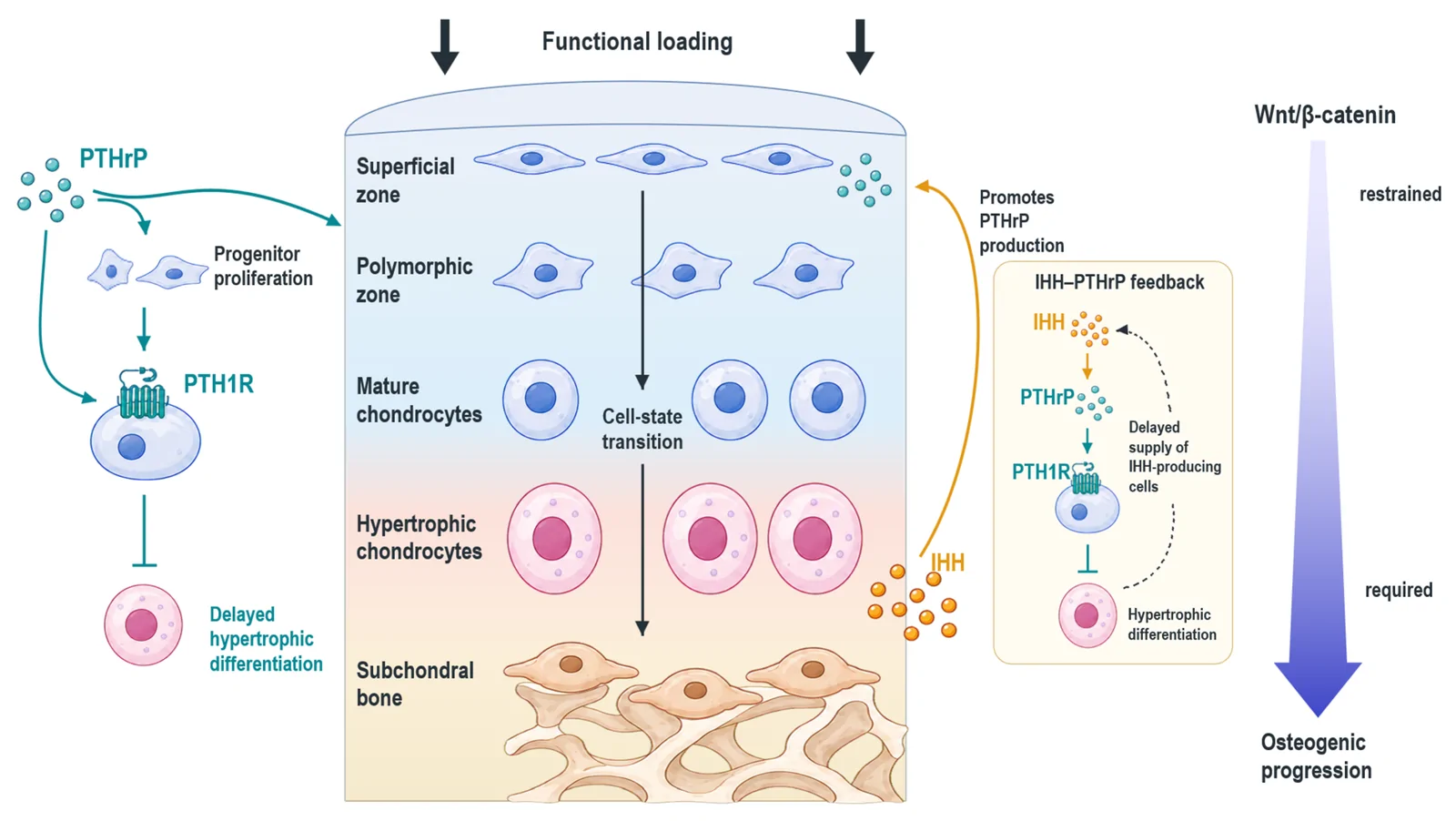
Spatial regulation of osteochondral cell-state progression by PTHrP, IHH and Wnt/β-catenin signalling. The central column depicts the superficial zone, polymorphic zone, mature and hypertrophic chondrocyte zones, and underlying subchondral bone. Functional loading acts on this zonally organised tissue. The PTHrP branch illustrates support for progenitor proliferation through its nuclear localisation segment and PTH1R-mediated restraint of hypertrophic differentiation. IHH produced by prehypertrophic and early hypertrophic chondrocytes contributes to PTHrP production in the upper cartilage. The feedback inset links PTHrP-dependent delay of hypertrophic differentiation to the subsequent supply of IHH-producing cells. The dashed arrow denotes this indirect feedback through cell-state progression. The purple gradient schematically represents restrained Wnt/β-catenin activity near the surface and its requirement during hypertrophic maturation and osteogenic progression. Arrowheads indicate the direction of the proposed regulatory effects; blunt-ended lines indicate restraint of hypertrophic differentiation. PTHrP, parathyroid hormone-related protein; PTH1R, parathyroid hormone 1 receptor; IHH, Indian hedgehog.

Together, these developmental pathways coordinate progenitor maintenance and proliferation with chondrocyte maturation and osteogenic progression (Figure 2).

### 3.4. Mechanical Regulation of PTHrP–IHH and Wnt/β-Catenin Signalling in TMJ Cartilage

Mechanical stimulation generated by jaw activity helps maintain PTHrP–IHH signalling in the growing mandibular condyle. This dependence is already present before birth. Restricting jaw movement in mouse embryos reduces IHH, PTHrP, PTCH1, and PTH1R expression and decreases cell proliferation and cartilage formation [79]. Similar changes occur after birth when masticatory loading is reduced. Across soft-diet, incisor-trimming and masseter-atrophy models, reductions in PTHrP and IHH expression are associated with thinner condylar cartilage and impaired subchondral bone development [51,54,80]. Together, these findings indicate that jaw activity provides mechanical input that helps sustain PTHrP–IHH signalling during condylar growth. Changes in the mechanical environment alter PTHrP–IHH pathway activity and thereby modify the pattern of condylar growth. In mandibular advancement models, increased IHH expression is associated with greater proliferation of mesenchymal cells, whereas increased PTHrP expression is associated with delayed hypertrophic maturation and enhanced cartilage formation [81,82]. Stepwise advancement produces repeated increases in IHH and PTHrP expression, whereas a greater magnitude of advancement elicits stronger increases [83,84,85]. Static pressure increases the expression of both PTHrP and IHH in cultured rabbit mandibular condylar chondrocytes [86]. These findings suggest that appropriate mechanical input modulates PTHrP–IHH signalling to coordinate cell proliferation, chondrocyte maturation and cartilage formation in the growing mandibular condyle.

Two signalling mechanisms have been reported to mediate the effects of mechanical stimuli on PTHrP expression in superficial-zone cells. In cultured rat superficial-zone chondrocytes, fluid-flow shear stress increases PTHrP production through the calcium-sensing receptor (CaSR) [63], whereas negative pressure increases PTHrP production through tuberous sclerosis complex 1 (TSC1)–mechanistic target of rapamycin (mTOR) signalling [64]. IHH expression is also mechanically responsive. When chondrocytes from superficial- and deep-zone cartilage were cultured separately, fluid-flow shear stress increased PTHrP expression in superficial-zone cells, whereas it increased IHH expression in deep-zone chondrocytes and decreased PTH1R expression [87].

Canonical Wnt/β-catenin signalling responds to changes in the mechanical environment during joint development. In the cartilaginous jaw joint of developing zebrafish, loss of muscle activity reduces Wnt/β-catenin activity in regions that are normally subjected to high mechanical strain. Reduced muscle activity also decreases cell proliferation and migration. Experimental manipulation of Wnt/β-catenin signalling further shows that this pathway acts downstream of mechanical activity and is required for normal jaw joint formation and chondrocyte maturation [13]. A similar response occurs in developing mouse limb joints. Loss of muscle activity reduces Wnt/β-catenin activity at the developing joint, whereas activity remains detectable in nearby perichondrial and subchondral cells [88]. In mandibular condylar cartilage, the direction of the Wnt response depends on the loading conditions. In a rat model of sustained posterior–superior compressive loading of the TMJ, β-catenin levels decrease, and GSK3β levels increase. These changes are accompanied by reduced chondrocyte proliferation and loss of cartilage matrix [89]. By contrast, models involving an increased occlusal vertical dimension or excessive forward positioning of the mandible activate β-catenin signalling [90,91,92]. In both models, the expression of Wnt antagonists, including secreted frizzled-related proteins and sclerostin, is reduced, and mandibular growth is disturbed, leading to degeneration of the condylar cartilage and subchondral bone [90,91,92]. Mechanical changes regulate Wnt/β-catenin signalling partly by altering β-catenin stability. In primary mandibular condylar chondrocytes, static compression reduces β-catenin phosphorylation at degradation-associated sites and increases total β-catenin, indicating that compression limits its degradation [90]. In mandibular condylar cartilage, altered occlusal loading in a UAC rat model increases the levels of connexin 43 (CX43), a hemichannel-forming protein, and β-catenin [93]. These findings suggest that appropriate mechanical loading is required to maintain the level and spatial distribution of Wnt/β-catenin activity required for normal condylar development. However, the molecular links connecting mechanosensitive pathways to PTHrP–IHH and Wnt/β-catenin signalling in condylar cartilage remain incompletely defined.

## 4. Disruption of Load-Responsive Osteochondral Fate: Progressive Degeneration and Failure of Progenitor Renewal

### 4.1. From Adaptation to Progressive Degeneration

Persistence of abnormal loading drives a transition from potentially reversible changes to progressive degeneration in mandibular condylar cartilage and subchondral bone. Progressive cartilage damage is observed across several experimental models of abnormal loading. In growing rats, sustained UAC progressively disrupts the normal zonal organisation of the condylar cartilage and promotes hypertrophic differentiation of chondrocytes and cartilage calcification [25]. More direct evidence for the progressive loss of reversibility comes from unloading studies. In models of sustained mouth opening and mandibular displacement, the degenerative histological changes induced by shorter periods of loading regress substantially after load removal, whereas longer exposure results in progressively less recovery [94,95]. The progressive changes include a transition from an early regional increase in cell proliferation to apoptosis, matrix loss, and surface disruption, as observed in the mouth-opening model [94]. In another rat model, an appliance was used to hold the mandible in a displaced position. One month after appliance removal, rats exposed to two weeks of mandibular displacement showed variable histological recovery, whereas those exposed for four weeks showed limited recovery [95]. Thus, although these models alter condylar loading in different ways, they consistently show that damage becomes more difficult to reverse as abnormal loading persists. The initial adaptive response gives way to degeneration. Taken together, these models suggest that persistent abnormal loading shifts condylar responses from adaptation towards degeneration, while progressively limiting the capacity for recovery after load removal (Table 1).

### 4.2. Imbalance Between Progenitor Replenishment and Mature Differentiation

Alongside enhanced hypertrophic differentiation in deep-zone cartilage induced by aberrant dental occlusion, superficial-zone progenitors are activated during the early stages of experimental temporomandibular joint osteoarthritis (TMJOA). These progenitors progressively differentiate to replenish cells in the deeper zones of degenerating cartilage. However, prolonged injury progressively reduces the superficial-zone progenitor population. Four weeks after UAC, the total population of genetically labelled *Sox9*-lineage cells declines despite increased Ki-67 labelling among the remaining cells. This decline occurs alongside increased proportions of *Sox9*-lineage cells expressing type X collagen and osteocalcin. By seven weeks, Ki-67 labelling declines, and the number of *Sox9*-lineage cells is lower than that in age-matched controls [99]. As degeneration progresses, superficial-zone progenitor populations identified by *Gli1* or *Sox9* lineage tracing decrease across experimental models [40,99]. The number of progenitors positive for the surface marker CD90 also decreases [98]. These findings suggest that accelerated lineage progression eventually outpaces progenitor renewal, progressively depleting the progenitor pool available for cartilage maintenance [99]. In the model of unilateral partial discectomy, cartilage thickness, the numbers of 5-ethynyl-2′-deoxyuridine (EdU)-positive and type X collagen-positive cells, and the proportion of *Gli1*-lineage cells increase one week after the operation [40]. Nearly 60% of *Gli1*-lineage cells expressed type X collagen, indicating that early lineage expansion was accompanied by accelerated hypertrophic differentiation [40]. By 12–16 weeks after surgical destabilization, the superficial zone is also sparsely populated, indicating a broader reduction in superficial-zone cells [100]. Overall, aberrant loading initially activates superficial-zone progenitors and transiently expands their population, while accelerating the progression of a subset towards chondrogenic and hypertrophic differentiation. With sustained injury, lineage progression outpaces progenitor renewal, progressively depleting the superficial-zone cell pool available for cartilage maintenance.

The responses of the superficial-zone progenitor population to aberrant loading are regulated by the distinct actions of PTHrP and β-catenin. The NLS of PTHrP mainly supports progenitor proliferation [63,64], whereas secreted PTHrP acting through PTH1R mainly serves as a molecular brake on hypertrophic maturation [58,87]. β-catenin contributes mainly to lineage progression by promoting the differentiation of progenitors towards chondrogenic and hypertrophic fates [40]. Early proliferation can therefore enlarge the superficial-zone progenitor population, while β-catenin activity simultaneously directs a subset of this population towards hypertrophy. As TMJOA progresses, PTH1R expression is reduced mainly because differentiated chondrocytes are lost. Cartilage thinning continues even though PTHrP levels remain elevated [87,101]. With persistent β-catenin-driven progression and insufficient restraint by PTHrP–PTH1R signalling, more progenitors progress towards hypertrophic differentiation, and subsequently either adopt a bone-cell fate or die. The superficial-zone progenitor pool is thereby progressively depleted.

### 4.3. Spatial Matrix Remodelling Alters Chondrocyte Function and Fate

Matrix remodelling in osteoarthritic cartilage is spatially heterogeneous rather than a uniform shift. UAC promotes calcification in deep-zone cartilage and subchondral cortical bone formation, therefore increasing stiffness at the osteochondral interface [102]. By contrast, in the main body of UAC-treated TMJ cartilage, significant proteoglycan loss is associated with a lower elastic modulus, indicating cartilage softening [103]. Nanoindentation likewise reveals a lower elastic modulus in the superficial zone of mandibular condylar cartilage in rats subjected to UAC [104]. Comparable superficial weakening has been observed in canine and human knee osteoarthritis, where collagen disorganisation and proteoglycan loss accompany reduced surface mechanical properties [105,106,107].

Within the superficial zone, loss of matrix stiffness may compromise the proliferative and reparative functions of resident progenitors. Reduced levels of type V collagen lower condylar cartilage stiffness and decrease the proliferation of superficial-zone progenitor cells [108]. Type V collagen deficiency also aggravates condylar cartilage degeneration under UAC-induced loading [109]. Matrix softening suppresses phosphoinositide 3-kinase (PI3K)–protein kinase B (AKT) signalling, thereby reducing the nuclear localisation of cAMP-responsive element-binding protein 5 (CREB5) and the expression of phospholipid phosphatase 3 (PLPP3). The resulting reduction in PLPP3 expression impairs mitochondrial respiration, superficial-zone cell migration and the secretion of fibronectin and lubricin. PLPP3 overexpression partially restores these functions and reduces cartilage damage [104].

Local matrix stiffness biases chondrocyte fate. Mandibular condylar chondrocytes cultured on stiffer substrates exhibit enhanced osteogenic differentiation, providing direct evidence that substrate stiffness can promote osteogenic differentiation in these cells [110]. Lineage tracing further shows increases in the proportions of DMP1-positive cells among genetically labelled *Sox9*- and *Col10a1*-lineage descendants in UAC-treated cartilage, consistent with enhanced osteogenic conversion in deep-zone cartilage, where UAC enhances calcification and stiffness [103]. The proportions of adiponectin-positive cells among *Sox9*- and *Col10a1*-lineage descendants also increase [103]. Loss of aggrecan reduces osmotic swelling pressure and may therefore partly explain tissue softening. Another potential contributor is lipid-droplet accumulation [111]. UAC in vivo and fluid-flow shear stress or cyclic tensile strain in vitro disturb chondrocyte microtubules, causing intracellular lipid-droplet accumulation. The chondrocytes then exhibit adipogenic features, which may compromise their chondrogenic phenotype and contribute to tissue softening [112,113,114]. Third, as observed in controlled mesenchymal stromal cell culture systems, soft substrates favour adipogenic differentiation, whereas stiffer substrates favour osteogenic commitment, partly through stiffness-dependent regulation of cytoskeletal tension and the activity of Yes-associated protein (YAP) and WW domain-containing transcription regulator protein 1 (TAZ) [115,116,117]. Overall, a stiff environment favours osteogenic conversion, whereas a soft matrix favours adipogenic differentiation. Together, calcification-associated stiffening near the osteochondral interface and softening of the main cartilage body associated with aggrecan loss, reduced osmotic swelling pressure, and lipid accumulation may contribute to spatially heterogeneous matrix remodelling under aberrant loading.

### 4.4. Terminal Chondrocyte Differentiation Converges with Subchondral Bone Remodelling at the Osteochondral Interface

The osteochondral interface is a transitional region connecting articular cartilage to the underlying bone. It comprises calcified cartilage and subchondral bone [102]. In UAC-induced TMJOA, both sides undergo spatially divergent remodelling. Calcified cartilage and subchondral cortical bone expand and stiffen the interface, whereas the deeper subchondral trabecular bone can simultaneously undergo bone loss [102,118]. Thus, interface thickening and stiffening reflect concurrent but non-uniform remodelling of cartilage and bone rather than a generalised increase in subchondral bone mass.

On the calcified cartilage side, UAC shifts deep-zone chondrocytes towards an alkaline phosphatase-positive terminal phenotype as enhanced IHH activity outweighs the weakened PTHrP–PTH1R restraint [87]. Matrix-vesicle production and basic calcium phosphate (BCP)-like mineral deposition progressively increase [56]. Mechanical stimulation also alters chondrocyte-derived exosomes, allowing a pro-calcific molecular programme to spread through the surrounding matrix [119]. Once formed, BCP crystals increase matrix-degrading enzyme levels and suppress matrix synthesis, allowing mineral deposition to accelerate local cartilage breakdown [56]. In osteoarthritic cartilage from other joints, BCP crystals concentrate WNT3A in the pericellular matrix and thereby stabilise β-catenin [120]. In UAC-induced TMJOA, Wnt/β-catenin signalling is also activated in mandibular condylar cartilage [93]. Sustained β-catenin activation driven by Acan-CreERT2-mediated recombination in TMJ chondrocytes causes deep-zone hypertrophy and chondrocyte loss, followed by woven bone formation within hypertrophic cartilage [121]. These findings suggest that established BCP deposits may reinforce β-catenin signalling in adjacent chondrocytes, extending interface remodelling beyond cartilage calcification towards disorganised ossification. Terminal differentiation, mineral deposition and β-catenin-associated ectopic ossification therefore progressively reshape the calcified cartilage component of the osteochondral interface, although the BCP–β-catenin interaction has not yet been demonstrated directly in TMJ cartilage.

In the deeper subchondral trabecular bone, increased osteoclast activity produces net bone loss despite a concurrent increase in osteogenic activity. In joints subjected to UAC-induced loading, WNT5A signals through receptor tyrosine kinase-like orphan receptor 2 (ROR2) in bone marrow stromal cells. This pathway increases C-X-C motif chemokine ligand 12 (CXCL12) production, which recruits osteoclast precursors. It also increases receptor activator of nuclear factor-κB ligand (RANKL) production, which promotes their differentiation into mature osteoclasts [118]. Cartilage damage provides an additional source of osteoclast-recruiting signals. In monosodium iodoacetate-induced TMJOA, p38 mitogen-activated protein kinase signalling is activated in apoptotic chondrocytes. This promotes the release of C-X3-C motif chemokine ligand 1 (CX3CL1). CX3CL1 attracts osteoclast precursors bearing its receptor, C-X3-C motif chemokine receptor 1 (CX3CR1), to the bone beneath the damaged cartilage [122]. This provides a mechanistic link between chondrocyte loss and osteoclast recruitment at the osteochondral junction. In partial-discectomy models, Hedgehog signalling promotes the expansion and abnormal osteogenic differentiation of *Gli1*-lineage progenitors, whereas *Ctnnb1* deletion in this lineage reduces pathological bone formation and ectopic mineralisation [123,124]. Increased osteogenic activity therefore does not prevent net loss in the deeper trabecular region.

**Figure 3:**
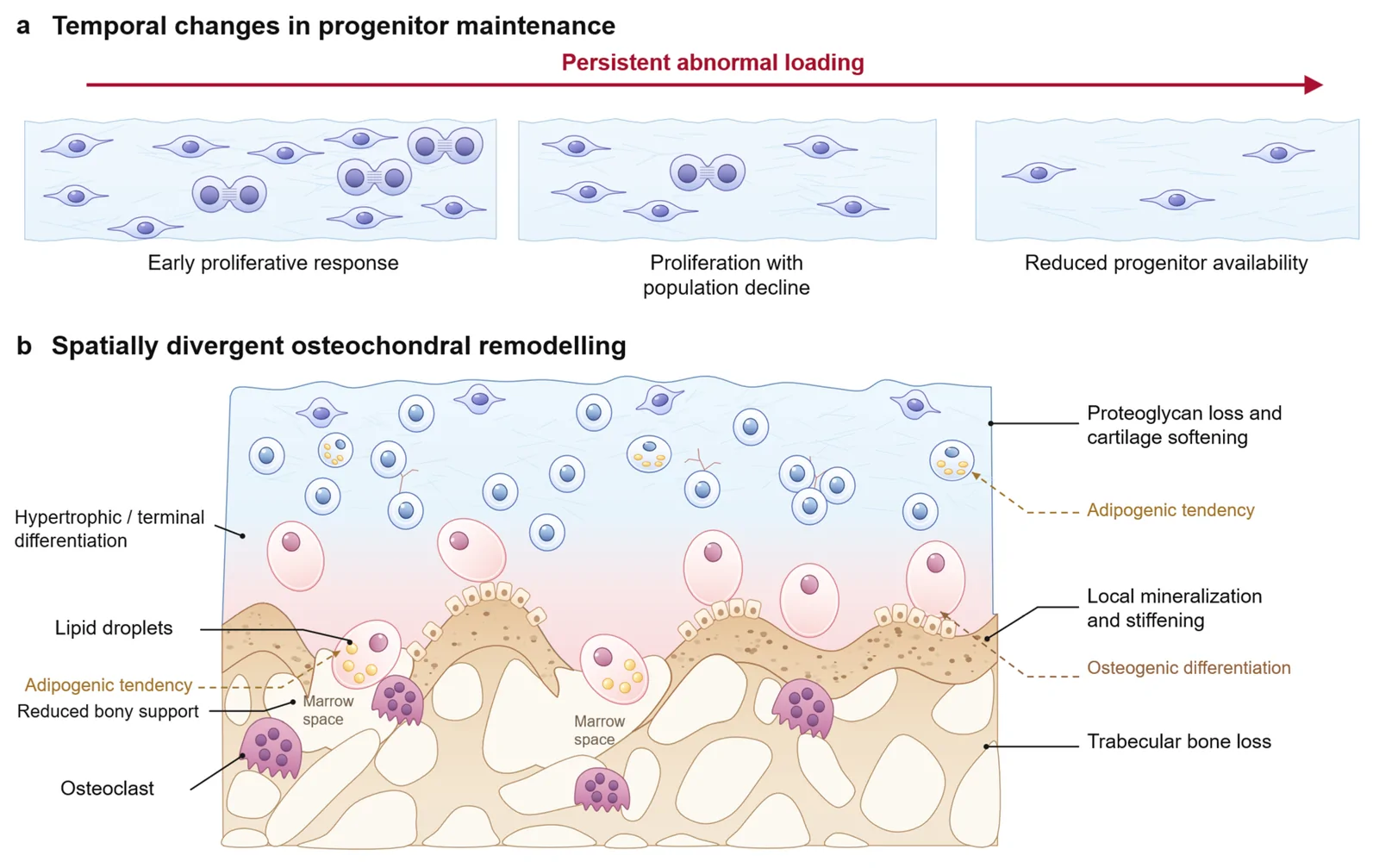
Temporal changes in progenitor maintenance and spatially divergent osteochondral remodelling under persistent abnormal loading. (**a**) Persistent abnormal loading initially induces a proliferative response in superficial-zone progenitors. Continued loading is accompanied by proliferation among the remaining cells but a net decline in the progenitor population, followed by reduced progenitor availability. (**b**) Degenerating cartilage exhibits matrix loss, focal microfissures and tissue softening. Mature chondrocytes remain above a disorganised, vertically staggered population of hypertrophic chondrocytes, illustrating accelerated hypertrophic and terminal differentiation. Lipid droplets are shown in selected chondrocytes and indicate focal intracellular lipid accumulation rather than completed adipocyte differentiation. The irregular osteochondral interface depicts increased mineralisation together with focal osteoblast- and osteoclast-associated remodelling. Interface thickening and stiffening may coexist with osteoclast-mediated loss of the deeper trabecular bone.

Together, these findings support a conceptual model in which persistent abnormal loading initially elicits a proliferative response but progressively reduces superficial-zone progenitor availability. Accelerated hypertrophic differentiation, focal lipid-droplet accumulation and cartilage matrix damage occur alongside spatially divergent osteochondral remodelling, characterised by cartilage softening, interface mineralisation and deeper trabecular bone loss (Figure 3).

## 5. Restoration of Load-Responsive Tissue Maintenance Through Staged Interventions

**Figure 4:**
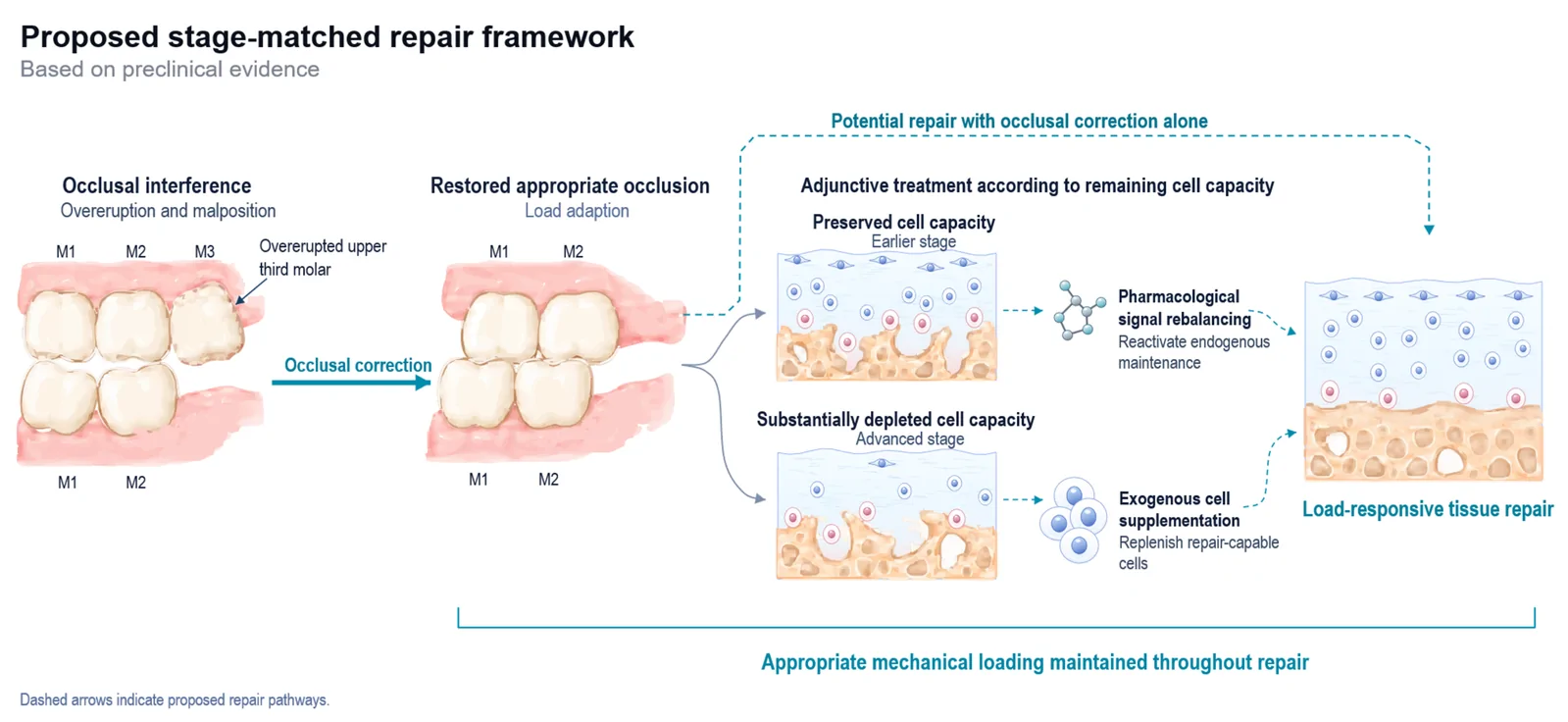
Proposed stage-matched repair framework for temporomandibular joint osteochondral degeneration. Persistent abnormal occlusal loading provides the starting condition for the framework. Modulation of an identified source of abnormal loading is proposed to establish a mechanical environment compatible with repair. Subsequent strategies are considered according to the remaining osteochondral repair capacity. When responsive resident cells are preserved, pathway-directed interventions may support endogenous tissue maintenance. When this capacity is substantially depleted, exogenous cell supplementation may provide additional reparative support. The early and advanced stages shown here are conceptual categories based on preclinical observations; their use for treatment selection requires validated measures of residual repair capacity. M1, M2 and M3 denote the first, second and third molars, respectively.

Restoration of tissue maintenance in TMJOA should begin with establishing an appropriate occlusal loading environment, because neither molecular intervention nor transplanted cells are likely to sustain repair under continued mechanical disturbance. Once abnormal loading and correctable structural factors have been addressed, biological treatment can be selected according to the remaining osteochondral cell population. At earlier stages, when sufficient endogenous cells remain, pharmacological intervention may rebalance dysregulated signalling and restore load-responsive tissue maintenance. In advanced degeneration with substantial cell loss, exogenous cells may instead be required to replenish the depleted repair population. Load adaptation is therefore the prerequisite for regeneration, whereas signalling modulation and cell supplementation represent biological strategies for earlier and later stages, respectively (Figure 4).

### 5.1. Correcting Pathological Occlusion to Restore a Load-Compatible Mechanical Environment

In patients with pathological occlusion that generates abnormal TMJ loading, correction of the pathological occlusal relationship should be the first therapeutic step. Here, pathological occlusion refers not simply to a morphological deviation from an ideal dental relationship, but to an occlusal condition that persistently produces excessive, asymmetric, or otherwise maladaptive force transmission during mandibular function. Because subsequent molecular or cell-based therapies remain exposed to the prevailing mechanical environment, this upstream source of pathological stimulation should be addressed initially to establish a stable and physiologically compatible occlusion that distributes force within the structural and load-bearing capacity of the TMJ.

Preclinical evidence indicates that pathological occlusal loading can cause TMJ osteochondral degeneration and that the resulting changes may be reversible. Experimentally imposed UAC and other abnormal occlusal relationships reduce chondrocyte numbers, disrupt proteoglycan and type II collagen production, increase cartilage catabolism, and promote osteoclast-mediated subchondral bone loss [57,125]. Removal of the abnormal prosthesis or restoration of physiological occlusal loading reverses many of these changes, including loss of cartilage thickness, reduced chondrocyte proliferation, matrix depletion, increased osteoclast numbers and subchondral bone loss [94,96,97,126,127]. Together, these findings indicate that correcting pathological occlusion can re-establish a mechanical environment permissive for endogenous osteochondral repair.

Correcting pathological occlusion should be the priority across disease stages, although the attainable biological outcome is likely to change. At an early stage, when degeneration is limited and responsive osteochondral cells remain, correction may allow substantial recovery of cartilage matrix and subchondral bone homeostasis [94,96,126]. With increasing age, prolonged exposure to abnormal loading and progressive depletion of superficial-zone progenitors, the capacity for structural recovery declines [98,128]. In advanced-stage TMJOA, correcting the pathological occlusion remains necessary because continued abnormal mechanical stimulation can worsen tissue damage even when the original osteochondral architecture may no longer be fully restorable. Neither pharmacological nor cell-based therapies are likely to achieve durable repair unless the pathological occlusion is corrected. Persistent abnormal occlusal loading may prevent the re-establishment of homeostatic signalling and undermine the reparative responses induced by the biological interventions.

### 5.2. Rebalancing Osteochondral Signalling to Mobilise Endogenous Repair

Rebalancing dysregulated osteochondral signalling may mobilise the remaining endogenous repair capacity of the diseased joint. The effectiveness of this strategy depends not only on the ligand or inhibitor delivered but also on the availability of target receptors, the preservation of responsive resident progenitors and the spatial organisation of the signalling niche [75,129,130].

Restoring PTHrP–PTH1R signalling may restrain premature chondrocyte hypertrophy and coordinate cartilage and subchondral bone remodelling. In a rat model of malocclusion-induced TMJOA, intermittent administration of parathyroid hormone (PTH) fragment 1–34 [PTH(1–34)] reduced cartilage degeneration, restored type II collagen expression and promoted subchondral bone formation [131]. Similar protective effects were observed in an ageing mouse model of TMJOA, in which PTH(1–34) reduced senescent cell accumulation and improved subchondral bone microarchitecture [132]. Supporting these findings, intermittent PTH(1–34) increased chondrocyte proliferation and cartilage thickness, reduced the expression of matrix metalloproteinase 13 (MMP-13) and a disintegrin and metalloproteinase with thrombospondin motifs 4 (ADAMTS-4), and enhanced subchondral bone formation in the mandibular condyles of adult mice [133]. These reparative effects depend at least partly on resident fibrocartilage stem cells (FCSCs). PTH promoted their proliferation and chondrogenic differentiation, whereas FCSC ablation in mice markedly reduced PTH-induced cartilage formation [130].

The therapeutic benefits of PTH(1–34) in preclinical TMJOA models were obtained with intensive repeated dosing [130,131,132]. Such frequent administration may be required because the pathological occlusal stimulus remains present. Abnormal occlusal loading is associated with progressive PTH1R downregulation, thereby reducing the responsiveness of the PTHrP–PTH1R axis [87,129]. Repeated ligand delivery may continuously activate the remaining receptors, but maintaining such frequent dosing through intra-articular administration is difficult to sustain clinically and does not itself establish a load-compatible biomechanical environment. Correction of the pathological occlusion should therefore precede PTH/PTHrP therapy, allowing pharmacological intervention to support repair rather than continually oppose an unresolved mechanical environment.

Excessive Wnt/β-catenin signalling can be therapeutically attenuated through local pathway modulation. Weekly intra-articular administration of sclerostin for seven weeks preserved the FCSC pool and promoted condylar cartilage regeneration in a rabbit TMJ injury model, with improvements in condylar morphology and proteoglycan distribution [38]. To prolong its local activity and reduce the injection burden, sclerostin was subsequently incorporated into StemJEL, a high-molecular-weight hyaluronic acid (HA) hydrogel. Two monthly injections of StemJEL restored chondrocyte identity, cartilage homeostasis, and joint function in rabbit and minipig models of post-traumatic TMJOA [75]. Therapeutic effects have also been achieved with other Wnt-directed interventions. A single intra-articular injection of the Wnt pathway modulator SM04690 protected condylar cartilage in both rabbit and rat partial-discectomy models and attenuated abnormal subchondral bone resorption and remodelling [134]. In malocclusion-induced TMJOA, local XAV-939 administration every other day for two weeks restored the expression of secreted frizzled-related proteins 3 and 4 (SFRP3 and SFRP4) and reduced cartilage degeneration [90]. A separate formulation encapsulated WNT16-loaded mesoporous silica nanospheres (MSNs) within HA hydrogels (HA/Wnt16@MSN). Sustained WNT16 delivery with this formulation restored cartilage integrity and suppressed osteoclastogenesis in a rat TMJOA model [135]. Collectively, these studies support local Wnt modulation as a promising strategy for protecting mandibular condylar cartilage and regulating subchondral bone remodelling. They also show that sustained-release formulations may substantially reduce the required frequency of intra-articular injections.

Collectively, these preclinical studies show that pathway-directed interventions can preserve resident progenitors, restore cartilage matrix metabolism and rebalance subchondral bone remodelling. Based on these observations, we propose addressing pathological loading before pharmacological intervention to establish a mechanical environment supportive of repair.

### 5.3. Considering Exogenous Cells When Endogenous Repair Is Insufficient

In TMJOA, accelerated differentiation of superficial-zone progenitors towards mature and terminal chondrocyte states progressively depletes the resident progenitor pool. Once this pool can no longer sustain osteochondral repair, rebalancing signalling may improve the behaviour of the remaining cells but cannot replenish the depleted progenitor pool [40,136]. Exogenous cell supplementation is therefore mechanistically suited to restoring the cellular basis for repair when endogenous capacity becomes insufficient.

Bone marrow-derived mesenchymal stromal cells (BMSCs) provide one potential exogenous cell source. In mice with UAC-induced TMJOA, weekly BMSC administration recruited transplanted cells to damaged cartilage. This recruitment involved CXCL12 and C-C motif chemokine ligand 5 (CCL5), acting through their receptors C-X-C motif chemokine receptor 4 (CXCR4) and C-C motif chemokine receptor 1 (CCR1), respectively. These cells differentiated into type II collagen-positive cells and attenuated cartilage degeneration and subchondral bone loss [137]. Subsequent work showed that BMSCs restored cartilage matrix primarily through matrix scavenging and replenishment rather than sustained proliferation. When BMSC administration was discontinued while UAC-induced abnormal loading persisted, the restored cartilage matrix deteriorated again [138]. Cartilage-protective, anti-inflammatory, and chondroregenerative effects have also been reported with mesenchymal stromal cells from other tissue sources across preclinical TMJOA models [139,140,141,142].

Cells derived from joint tissues may provide a cell source better adapted to the joint environment. Human TMJ FCSCs show SOX9-dependent chondrogenesis in vitro. Rat FCSC transplantation also supported cartilage repair in a rat condylar defect model. Reduced SOX9 expression in human TMJOA cartilage may restrict endogenous repair [143]. Similarly, CD90-positive chondrocytes from rodent TMJ cartilage or human osteoarthritic knee cartilage promoted osteochondral repair after transplantation into experimental UAC-induced TMJOA joints. Repeated administration produced greater structural repair than a single injection. However, the transplanted cells rapidly matured and differentiated when UAC was maintained [136]. Sustained repair therefore requires not only an adequate supply of cells but also a biomechanical environment that preserves their chondrogenic phenotype.

However, these findings derive mainly from animal studies, and their clinical applicability to human TMJOA remains uncertain (Table 2). Clinically validated criteria for assessing residual repair capacity are also lacking, limiting the use of this framework to guide treatment selection. In addition, terminology and identification criteria vary across studies, and marker-defined populations may only partially overlap without being equivalent. In this review, we retain the original study-specific nomenclature without treating these terms as interchangeable. The potential cell-group differences among the reports may lead to a deviation of the conclusion.

## 6. Conclusions

Mandibular condylar development, degeneration and regeneration are governed by a load-sensitive balance between progenitor renewal and chondrocyte maturation, coordinated through the dual modes of PTHrP signalling, together with IHH and Wnt/β-catenin signalling. In TMJOA, pathological loading disrupts these pathways, accelerates terminal differentiation and depletes the resident progenitor pool. We therefore propose a therapeutic strategy that begins by correcting pathological loading to restore a mechanically compatible environment, followed by modulation of these signalling pathways when endogenous repair capacity persists or by exogenous cell supplementation when the resident progenitor pool has been severely depleted.

## Figures and Tables

**Table 1:** Representative mechanical loading models of the mandibular condyle and responses to load modification.

Model and Animals	Loading Protocol and Duration	Molecular/Cellular Responses	Tissue Outcomes	Recovery After Load Modification	Ref.
**Reduced masticatory loading** Mice, 3 weeks old	Soft gel diet versus hard chow for 6 weeks	Reduced proliferation and cartilage matrix markers	Reduced mandibular growth and fewer chondrocyte-derived bone cells	Not assessed	[53]
**Unloading and reloading** Mice, 3 weeks old	Soft diet and incisor trimming for 2, 4 or 6 weeks; 2-week reloading after 4-week unloading	Reduced Indian hedgehog and cartilage matrix transcripts during unloading	Thinner cartilage, matrix loss and transient subchondral bone loss	After 2 weeks of reloading, cartilage width restored; thickness and matrix staining partly recovered	[54]
**Mandibular advancement** Rats, 5 weeks old	Continuous 2 or 4 mm advancement for up to 30 days	Indian hedgehog and parathyroid hormone-related protein (PTHrP) transcripts peaked at day 7	Greater new condylar bone formation with 4 mm advancement	Not assessed	[85]
**Bilateral anterior elevation (BAE)** Mice, 6 weeks old	Incisor appliances elevated occlusion for 3–11 weeks, depending on the study	Increased PTHrP expression, mechanistic target of rapamycin activity, and superficial-zone proliferation	More superficial-zone cells and thicker cartilage	Not assessed	[63,64]
**Sustained compression** Rats, 6 weeks old	Continuous loading at 80 g-force per side for 4 or 7 days	Reduced β-catenin and proliferation; early apoptosis	Cartilage thinning, matrix loss and trabecular damage	Not assessed	[89]
**Unilateral anterior crossbite** Mice, 6 weeks old	UAC removal after 3 weeks, or removal/replacement with BAE after 7, 11 or 15 weeks; 4-week follow-up after load modification	Increased matrix-degrading enzymes; reduced catabolic signals after load modification	Cartilage thinning, cell and matrix loss, and subchondral bone loss	Recovery remained partial after prolonged loading; BAE more effective than removal after 11–15 weeks	[96,97]
**Bilateral anterior crossbite** Mice, 6 or 28 weeks old	Loading for 3, 7 or 11 weeks; removal at week 7 with 4-week follow-up	Increased apoptosis and later reduced proliferation	More severe cartilage and bone degeneration in older mice	Greater recovery in younger mice; older mice showed no significant recovery of cartilage thickness or histological grade	[98]
**Mandibular shift** Growing rats	Approximately 2 mm lateral displacement for 1, 2 or 4 weeks; 4-week follow-up after removal	Reduced differentiation factors and cartilage matrix markers	Progressive cartilage loss, condylar deformation and subchondral bone changes	Substantial recovery after 1 week, variable after 2 weeks and limited after 4 weeks of loading	[95]
**Forced mouth opening** Mice, 8 weeks old	A 0.5-N force for 1 h/day for 3, 5, 10 or 20 days; 1-month follow-up after cessation	Early proliferation followed by apoptosis	Progressive cartilage damage, matrix loss and regional subchondral bone loss	Near-normal cartilage after 3–5 days of loading; incomplete recovery after 10–20 days	[94]

**Table 2:** Representative pharmacological and cell-based interventions in mandibular condyle models.

Intervention and Model	Administration and Regimen	Main Outcomes and Limitations	Ref.
**Local parathyroid hormone (PTH)** Rats, 6 weeks old; partial discectomy-induced osteoarthritis	Intra-articular PTH(1–34), 40 µL at 10 nM, every 3 days for 4 or 6 weeks; started 4 weeks after surgery	Increased cartilage thickness, matrix staining and subchondral bone mass	[130]
**Systemic PTH(1–34)** Rats, 160–180 g; malocclusion-induced osteoarthritis	Subcutaneous, 40 µg/kg daily, 5 days/week for 4 weeks; started 30 days after induction	Reduced cartilage degradation and increased subchondral bone formation	[131]
**Sclerostin** Rabbits, 12 weeks old; disc perforation	Intra-articular, 100 µL at 100 ng/mL, weekly for 7 weeks; started 1 week after surgery	Preserved resident fibrocartilage stem cells and cartilage matrix. Joint biomechanics and subchondral bone outcomes were not evaluated	[38]
**StemJEL** Hyaluronic acid with sclerostin; rabbits and miniature pigs, 6 months old; disc perforation	Intra-articular, 2% hyaluronic acid with sclerostin (150 ng/mL); 100 µL in rabbits or 1 mL in pigs, at 1 and 2 months after surgery	Preserved chondrocyte identity and cartilage organisation; improved cartilage mechanical properties at 3 months after surgery	[75]
**XAV-939** Rats, 6 weeks old; increased occlusal vertical dimension	Local joint injection, 50 µL at 0.4 mM, every other day for 2 weeks; started 2 weeks after induction	Reduced cartilage thinning, matrix loss and inflammatory signals	[90]
**SM04690** Rabbits, 12 weeks old, and rats, 6 weeks old; partial discectomy	Single intra-articular injection 1 week after surgery: 1.75 µg/100 µL in rabbits or 0.3 µg/50 µL in rats	Reduced cartilage damage and abnormal subchondral bone remodelling; increased WNT16 expression at the study endpoint injection. Efficacy in advanced disease was not established	[134]
**WNT16 hydrogel** Hyaluronic acid–mesoporous silica nanoparticle formulation; rats, 6 weeks old; unilateral anterior crossbite (UAC)	Bilateral intra-articular injections: WNT16 100 ng/mL, hyaluronic acid 10 mg/mL and nanoparticles 100 µg/mL; weekly from 1 week after UAC	Reduced cartilage damage, osteophytes and subchondral bone loss at the reported 2-, 4- and 6-week endpoints	[135]
**Bone marrow mesenchymal stromal cells** Mice, 6 weeks old; UAC	Bilateral local joint injections, weekly for 4, 8 or 12 weeks, starting 3 weeks after UAC. Reported dose: 2000 cells/20 µL; per side versus bilateral total unspecified	Improved cartilage matrix and subchondral bone structure. Deep-zone deterioration resumed after treatment cessation under continued UAC [138]	[137,138]
**CD90-positive chondrocytes** Mice; UAC initiated at 6 weeks old	Bilateral local joint injections, 20 µL/side at 100,000 cells/mL, weekly for 4 weeks; started after 3 or 21 weeks of UAC	Improved cartilage thickness and cellularity; repeated dosing outperformed one injection. Continued UAC promoted transplanted-cell terminal differentiation.	[136]
**Fibrocartilage stem cells** Rats; 1.2 mm condylar cartilage defect	Single placement of 1 million cells in 25 µL into the defect during surgery; assessed after 4 weeks	Improved defect repair, cartilage organisation and histological scores. Acute focal defect model; applicability to chronic osteoarthritis remains uncertain	[143]

## Data Availability

No new data were created or analyzed in this study. Data sharing is not applicable.
